# Breeding and Genomic Approaches towards Development of Fusarium Wilt Resistance in Chickpea

**DOI:** 10.3390/life13040988

**Published:** 2023-04-11

**Authors:** Rakesh Kumar Yadav, Manoj Kumar Tripathi, Sushma Tiwari, Niraj Tripathi, Ruchi Asati, Vinod Patel, R. S. Sikarwar, Devendra K. Payasi

**Affiliations:** 1 Department of Genetics & Plant Breeding, College of Agriculture, Rajmata Vijayaraje Scindia Krishi Vishwa Vidyalaya, Gwalior 474002, India; 2 Department of Plant Molecular Biology & Biotechnology, College of Agriculture, Rajmata Vijayaraje Scindia Krishi Vishwa Vidyalaya, Gwalior 474002, India; 3 Directorate of Research Services, Jawaharlal Nehru Krishi Vishwa Vidyalaya, Jabalpur 482004, India; 4 Regional Agricultural Research Station, Sagar 470001, India

**Keywords:** Fusarium wilt, conventional breeding, molecular makers, QTLs, genomics, transcriptomics, metabolomics and proteomics

## Abstract

Chickpea is an important leguminous crop with potential to provide dietary proteins to both humans and animals. It also ameliorates soil nitrogen through biological nitrogen fixation. The crop is affected by an array of biotic and abiotic factors. Among different biotic stresses, a major fungal disease called Fusarium wilt, caused by *Fusarium oxysporum* f. sp. ciceris (*FOC*), is responsible for low productivity in chickpea. To date, eight pathogenic races of *FOC* (race 0, 1A, and 1B/C, 2-6) have been reported worldwide. The development of resistant cultivars using different conventional breeding methods is very time consuming and depends upon the environment. Modern technologies can improve conventional methods to solve these major constraints. Understanding the molecular response of chickpea to Fusarium wilt can help to provide effective management strategies. The identification of molecular markers closely linked to genes/QTLs has provided great potential for chickpea improvement programs. Moreover, omics approaches, including transcriptomics, metabolomics, and proteomics give scientists a vast viewpoint of functional genomics. In this review, we will discuss the integration of all available strategies and provide comprehensive knowledge about chickpea plant defense against Fusarium wilt.

## 1. Introduction

Chickpea (*Cicer arietinum* L.) is a self-pollinating, annual diploid (2n = 2x = 16) species with a genome size of 738 Mb [1]. It is also referred to as gram, Bengal gram, Egyptian pea, garbanzo, or garbanzo bean [2]. It encourages biological nitrogen fixation, which boosts soil fertility. The family Fabaceae (Leguminosae), subfamily Faboideae (Papilionaceae), and tribe Cicereae make up the taxonomic hierarchy of chickpeas. There are nine annual species and roughly 34 perennial wild species [3]. The only annual species that is grown commercially is *Cicer arietinum* [4,5]. 

There are two varieties of grown chickpea: Kabuli and Desi. The Desi (microsperma) varieties of plant contain thick seed coats, pink blooms, and stems that are anthocyanin-pigmented [6], while the Kabuli (macrosperma) varieties of plant have white blooms, white- or beige- colored seeds with a ram’s head shape, a smooth seed surface with a thin seed coat and an absence of anthocyanin coloration on the stem [5]. Every year, more than 2.3 million tons of chickpeas are imported to supplement the needs of many nations of the world that are unable to produce a large enough quantity to satisfy their domestic demand [7]. The top exporters are Australia, Argentina, and Canada. The Kabuli variety of chickpea is grown extensively in West Asia, North Africa, North America, and Europe [7].

Chickpea seeds are nutrient-dense foods that have a high protein content and include dietary elements such as calcium, iron, and phosphorus [8]. The seeds include modest amounts of thiamin, vitamin B_6_, magnesium, and zinc, as well. They are beneficial in the management of various serious human diseases such as diabetes, cardiovascular disease, and digestive disorders [9,10]. Excluding sulfur-containing amino acids, chickpea seeds contain several important amino acids. On the surface, chickpea grains contain: 17.1% protein, 60.9% carbs, 5.3% fats, 3% minerals, and 3.9% crude fiber [11]. The measurement of free proline levels is a helpful indicator for assessing plant physiological condition and stress [12]. Despite having just trace levels of lipids, chickpea contains unsaturated fatty acids such as linoleic and oleic acids [13]. Essential sterols, viz., stigmasterol, campesterol, and sitosterol, are also found in chickpea oil [14]. Despite these benefits, numerous biotic factors, such as Fusarium wilt and Ascochyta blight diseases and the insect pest known as the pod borer, along with abiotic challenges, such as drought, salinity, and heat, have a significant influence on yields of chickpea [15]. By alleviating these challenges, chickpea productivity can be increased. While efforts have been made using an array of conventional methods [16,17,18], there is significant potential for advancement when they are combined with molecular methods, such as genomics-assisted breeding [19,20]. Chickpea breeding aims to increase production by pyramiding genes for drought, cold, salinity, fungal, and pod borer resistance / tolerance into superior chickpea genotypes [21].

Since chickpeas are self-pollinated, the target feature, i.e., wilt resistance, may be easily incorporated in the desired genotype after successful introgression [22]. Backcross, recombination breeding, and other traditional approaches are equally effective in developing cultivars with wilt resistance [23]. Several Fusarium wilt (FW) resistant donors and cultivars have been identified and released in chickpea as a result of straightforward field screening and selection under wilt-diseased plots [24]. Numerous crosses may be generated to develop segregating populations, which is a crucial prerequisite for undertaking a successful crop improvement program [25]. However, the mapping of populations in chickpea for the purpose of identifying targeted genes and constructing linkage maps is challenging due to the requirement of large numbers of plants in the mapping population [26,27]. To overcome these challenges, researchers are using advanced breeding technologies to identify targeted genes and the mechanisms of their interaction with each other or with environmental conditions [28]. The combination of modern approaches with traditional breeding technology is useful in the analysis of the mechanism of Fusarium wilt resistance, as well. The prime goal of traditional breeding in legumes is to increase yield. 

As a result, modern breeding techniques can be employed to enhance crop yields [29]. However, this notion has begun to change in the last decade due to improved novel techniques and the associated decreasing cost [24]. As a result of the crop’s economic importance, research on chickpea genomics has recently surged, and a wealth of genomic materials, including molecular markers and linkage maps, ESTs, and NGS-based transcriptomes, have become readily available [28].

Among advanced technologies, marker-assisted selection (MAS) has helped in targeting desirable genes [30]. Markers have demonstrated their role in enhancing selection efficiency and creating novel cultivars [31,32]. Recently, the integration of several “omics” methods has been developed into effective solutions for plant systems with the development of superior cultivars [33,34]. In order to address a variety of biological concerns, second-generation sequencing [35,36,37] is currently extensively employed. The genetic resources for chickpeas have, however, significantly enhanced in recent years with the applications of next-generation sequencing initiatives and their application in genomics research [38,39,40]. The current review aims to summarize all the advancements made, obstacles encountered thus far, and prospects for future advancements in chickpea Fusarium wilt resistance.

## 2. Fusarium Wilt

**Figure 1:**
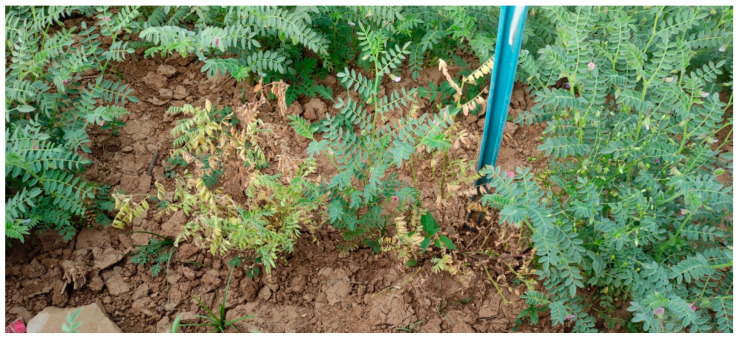
Fusarium wilt-infected chickpea plants.

Fusarium wilt, caused by *Fusarium oxysporum* f. sp. ciceri, is important due to its severe effects on the yield of chickpea [41,42]. It is most common in hot, dry regions and can result in annual output losses of up to 10% to 15%, with epidemics leading to yield losses of up to 100% [43,44]. According to Verma et al. [23], it has eight different types of pathogenic races and pathotypes, which may be a reason for its pathogenic diversity. Based on their ability to produce unusual symptoms, the races are categorized. Major plant symptoms associated with Fusarium wilt disease infection (Figure 1) include yellowing and wilting [45]. The ability of the races to evoke separate reactions that result in two different sorts of symptoms—yellowing and wilting—sets them apart from one another. More dangerous than yellowing syndrome is with erring syndrome [46].

In six continents, 32 countries are affected by chickpea wilt [47]. Butler originally described this disease in India in 1918, but it was not until Padwick accurately identified its cause in 1940 that it was fully understood [48]. Different levels of yield losses have been documented in chickpea due to FW (40% [49] and 77–94% [50]). In the case of “late wilt”, dropping petioles and leaf yellowing symptoms appear during the podding stage, resulting in yield losses of 24–65 percent. The yellowing pathotype of *F. oxysporum* f. sp. ciceris causes a disease condition in chickpeas that is comparable to that of *F. redolens* (*FOC*). Because it is challenging to distinguish between *Fusarium redolens* and *F. oxysporum* using morphology-based diagnosis, and because the two species affect chickpea in ways that are similar, the use of molecular techniques may be required in the efficient identification of the Fusarium pathotype in chickpea [50,51].

**Figure 2:**
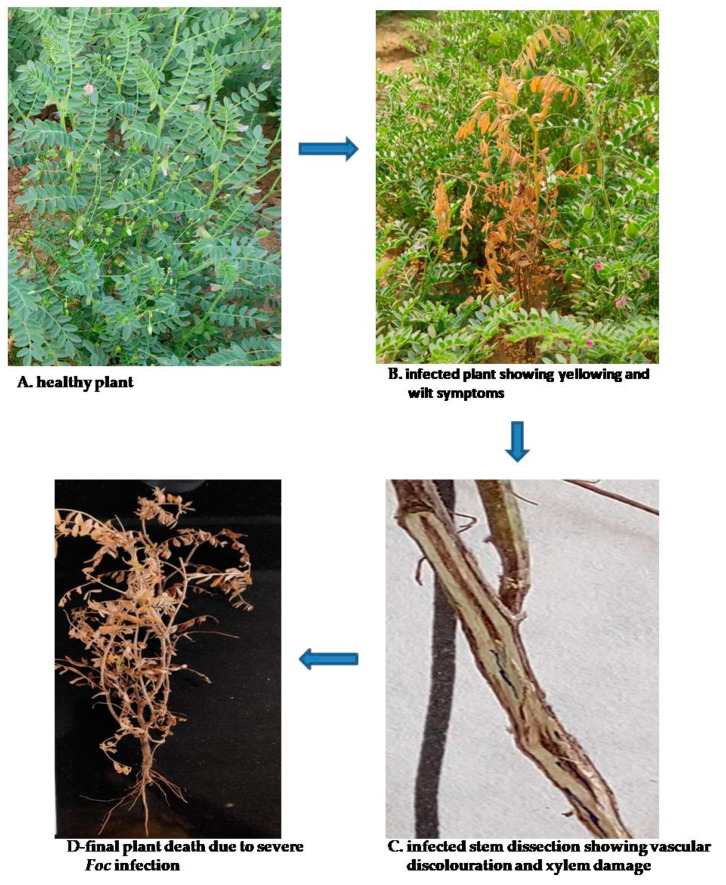
Sequence of Fusarium wilt infection in chickpea plants.

The amount of yield loss due to wilt disease in chickpea depends on the agro-climatic conditions of the region. Sometimes, the wilt disease becomes more dangerous, resulting in severe damage (Figure 2) and yield failure [52]. Fusarium wilt is a disease that spreads through the soil. It has an array of mechanisms of transmission, such as through contaminated plant wastes (leaf, root, and stem), soil and seeds, macroconidia, mycelium, and most frequently, chlamydospores [50,53].

The Indian subcontinent and areas where crops are cultivated in the spring and more regularly manifest under warm, dry growing circumstances are more troubled by Fusarium wilt [27]. Fungicidal seed coats provide protection against infection transmitted by seeds, but because the pathogen is persistent in soil, the best way to eradicate the infection is through host resistance. The pathogen gains access to the vascular bundles of the chickpea plants and blocks or lowers water intake to the foliage. The infected plants eventually wilt and die [28]. The causes include a buildup of fungus mycelium in the xylem and/or the production of toxins, host defense mechanisms such as the production of gels, gums, and tyloses, and vessel crushing brought on by the expansion of nearby parenchyma cells [54].

## 3. Genetics of Resistance to Fusarium Wilt

The *Fusarium oxysporum* f. sp. ciceris (*FOC*) pathogenies known to possess great pathogenic diversity that is classified into different pathogenic races, including races 0 and 1A, 1B/C, 2, 3, 4, 5, and 6. Additionally, two categories of FW symptoms have been identified: early yellowing and late wilting [55,56]. Additionally, researchers have also looked at the genetics of races 1A, 2, 3, 4, and 5 [57]. The symptomatic wilting pathotype induces quick and severe chlorosis, flaccidity, vascular discoloration, and early plant death, mostly in races 1A, 2, 3, 4, 5, and 6 [55], whereas the symptomatic yellowing pathotype instigates slow foliar yellowing, vascular discoloration, and late plant death in races 0 and1B/C [56,57].

It has been documented that chickpea resistance to Fusarium wilt can be either monogenic or oligogenic (Table 1) depending on the source or race of the resistance [57]. Three distinct genes (h_1_, h_2_, and H_3_) independently govern resistance to race 1A, according to early investigations on *FOC* [58]. Late wilting resistance can be conferred by any one of these three genes, but total resistance can be conferred by any two of these genes (h_1_h_2_, h_1_H_3_, or h_2_H_3_) [59]. While resistance to race 3 has been proven to be monogenic, resistance to race 2 is controlled by a single recessive gene [60,61]. As stated in earlier studies, race 4 resistance is recessive and digenic, but race 5 resistance is governed by a single gene [62].

Geographical classifications of the pathogenic races of *FOC* have been made. Indian, Mediterranean, and American populations of race 1A have been documented [63]. In addition, race 4 has been documented in Ethiopia, India, and Iraq [64,65]. Races 0,1B/C,5, and 6 are most common in the Mediterranean Basin and California (USA) [66], while races 2 and 3 have been observed in Ethiopia, India, and Turkey [50].

**Table 1:** Genetics of resistance to races of the chickpea wilt *Fusarium oxysporum* f. sp. ciceris.

Fusarium Race	Name of Resistance Gene	Number and Nature of Wilt Resistance Gene	Effect of Resistance Gene on Wilting	Symptoms	References
0	*FOC-01/FOC-01*	Monogenic or digenic	Complete resistance	Yellowing	[26]
	*FOC-02/FOC-02*				
1A	*h1 (syn FOC-1)*	Trigenic	Late wilting	Wilting	[57]
	*h2*		Late wilting		
	*H3*		Late wilting		
1B/C	*-*	-	-	Yellowing	[63]
2	*FOC-2*	Monogenic	Complete resistance	Wilting	[27]
3	*FOC-3/FOC-3*	Monogenic	Complete resistance	Wilting	[62]
4	*FOC-4*	Monogenic recessive	Complete resistance	Wilting	[27]
5	*FOC-5/FOC-5*	Monogenic	Complete resistance	Wilting	[67]
6	-	-	-	Wilting	[63]

## 4. Breeding Methods Employed for Fusarium Wilt Resistance in Chickpea

Higher and more consistent yields are the main objectives of chickpea breeding programs [15]. According to an investigation conducted by Srivastava et al. [68], chickpea resistance to Fusarium wilt may be either monogenic or oligogenic, depending on the resistance source or race. The selection of plants for characteristics and disease resistance is the second most important step in a breeding program involving evaluation of the plant for commercial production.

Breeding programs are dependent upon the magnitude of genetic variation present in the population. The type and degree of diversity influence a breeding strategy’s efficacy. Even though the disease is soil-borne, chemical control is ineffective and impractical to use [69]. Utilizing host plant resistance is the most reliable strategy for solving the problem. Several sources of chickpea resistance to Fusarium wilt have been found in the past. These resistance sources have been identified using different methods, including a wilt-diseased plot in the field and hot spot location screening, as well as greenhouse and laboratory procedures [70,71,72]. The majority of these methods were employed in resistance breeding programs at the National Agricultural Research System (NARS) and International Crops Research Institute for the Semi-Arid Tropics (ICRISAT), which significantly increased chickpea productivity in semi-arid parts of Africa and Asia [73,74]. However, in these areas, substantial genetic diversity in the pathogen and GxE interaction have an impact on resistance durability. A variety of strategies, including the GGE billet technique, have been utilized in different studies to investigate the GxE interaction [75]. Utilizing biplot analysis of GxE data, it is now possible to graphically address many important aspects to develop a better understanding, including genotype stability, mean performance, discriminating ability, mega-environmental investigation, representativeness of the environment, and who-resistant-where pattern [76,77,78].

The process of using plants as a strategy involves gathering and analyzing genotypes from different sources in order to find suitable genotypes that are adapted to the local environment and have high productivity or any other desired specialized attribute [79]. As a result, the type of material introduced determines whether plant introductions are successful. Genes must be fixed in breeding lines in order to create pure-line cultivars. The initial selection process that uses landraces is the simplest and is known as mass or pure-line selection. Crossover programs and several iterations of pedigree and bulk approaches were employed to manage segregating generations [79,80]. Through pure-line selection, the JG315 chickpea cultivar evolved resistance to Fusarium wilt in Madhya Pradesh, India. The JG 62 cultivar, in addition to race 0, is a variety that is very vulnerable to FW, whereas ICCV 05530 is a cultivar that is highly resistant to FW [81].

Most breeding operations for chickpeas use single-cross hybridization. Hybridization almost occurs within the same species of the genetically distinct Desi and Kabuli varieties [82]. To promote genetic diversity and introduce beneficial genes from wild *Cicer* spp. into cultivated species, interspecific crosses have been attempted. *FOC* race resistance has largely been found in the Desi germplasm and in wild *Cicer* spp. In fact, accessions of *C. bijigum*, *C. cuneatum*, and *C. judaicum* showed combined resistance against races 0 and 5, but accessions *C. canariense* and *C. chorassanicum* were found to be resistant to race 0 whenever vulnerable to race 5. Additionally, the *C. pinnatifidum* accessions evaluated were found to be vulnerable to race 5, whereas some were resistant to race 0 [83].

Various chickpea breeders have used traditional methodologies and breeding techniques, and the population has improved in terms of increased output, different resistance, and desired plant types. Regarding FW response, genetic heterogeneity in chickpea genotypes has been recorded [84]. In accordance with the earlier findings, resistant sources were identified against FW in both Kabuli (ICCV 2 and UC 15) and Desi types (FLIP 85-20C, FLIP 85-29C, and FLIP 85-30C). Numerous chickpea Fusarium wilt-resistant genotypes, including ICCV 98505, ICCV 07105, ICCV 07111, and ICCV 07305, were identified by Sharma et al [85] using GGE biplot analysis. Four Kabuli chickpea genotypes resistant to FW, including ICCV 2, ICCV 3, ICCV 4, and ICCV 5 (Table 2), were previously generated using the pedigree method. Crop breeders now have a range of more effective tools for resistance breeding owing to recent developments in legume genomic technologies. As a result, legume crops can now be improved using genomics to better withstand different biotic and abiotic challenges [86,87].

Pande et al. [70] found twenty-one accessions free from FW disease and twenty-five that were resistant during their study on the screening of chickpea genotypes against FW. In a separate study, genotypes JG 315, Avrodhi, DCP 92-3, JG 74, BG 372, and KWR 108 were found to be resistant to Fusarium wilt [87], while ICCV 05530 maintained its resistance against two FW races, viz., 1 and 3. Among these genotypes, JG 62 showed 89–100% wilt incidence against both FW races.

The use of nested association mapping (NAM) and multi-parent advanced generation intercross (MAGIC) populations is being developed in chickpea to make inter-crosses between multiple (4, 8, or 16) parental lines that originate from diverse regions. The creation of these crosses is possible through the balanced funnel crossing method, which recombines mosaics of founder parents, resulting in novel genotype and haplotype combinations [89]. At ICRISAT, a MAGIC population was created by mating cultivars and elite breeding lines, including ICC 4958, ICCV 10, JAKI 9218, JG 11, JG 130, JG 16, ICCV 97105, and ICCV 00108, with eight varied founder parents [73,85,88].

## 5. Screening Strategies to Identify Wilt-Resistant Genotypes

The utilization of host plant resistance (HPR) begins with the development of trustworthy and reproducible disease screening techniques to assess many germplasm accessions and breeding materials. It has been claimed that screening in the field and under controlled conditions (such as in greenhouse and lab settings) may help to identify resistant genotypes against FW [94]. However, there are some problems associated with maintaining uniform conditions for each plant during the screening of genotypes. So, it is important to develop a simple and efficient technique to screen chickpea genotypes for the identification of FW-resistant cultivars for future breeding programs. Generally, the following methods are applied for the screening of Fusarium wilt-resistant chickpea genotypes.

### 5.1. Field Screening

The most frequent and recurrently applied technique for identifying FW-resistant genotypes is the wilt-diseased plot (WDP) strategy. The primary advantage of the WDP technique is that it makes it possible to screen a vast array of genetic materials under field conditions [95]. Effective wilt-diseased plots for field and hot spot location screening, as well as greenhouse and laboratory methodologies and successful breeding programs, have all been created [96]. Assessing inoculum homogeneity in a plot involves planting test genotypes next to susceptible cultivars as an indicator line or checking susceptibility after every 2–4 test entries. The widely applied susceptibility checks for races 1 to 4 in India include “JG 62”, a twin-podded chickpea type that is extremely susceptible to all *FOC* races except race 0. The cultivar “JG 74” and the germplasm line “WR 315” (ICC 11322) of chickpeas are the two main sources of resistance. While the latter is resistant to all races but race 2, the prior is resistant to all *FOC* races except for race 3. The stepwise identification of host plant resistance to diseases has recently been revised by Pande et al. [89]. In order to screen many germplasm lines against FW, WDPs have been created at the International Center for Agricultural Research in the Dry Areas (ICARDA), ICRISAT, and NARS of countries that cultivate these crops.

Chickpea wilt has been investigated globally since the last decade of the 20th century using several methods. These efforts have involved the creation of multiple disease grading scales to calculate disease incidence and prevalence when evaluating new chickpea germplasm lines. Disease reactions are categorized based on the proportion of dead plants, whereas physiological maturity represents the reaction score of each genotype. To determine phenotypic resistance and susceptibility for race identification, different disease scoring scales are applied.

The six-point scale makes scoring simple (Table 3). Interpretation of the scale is as follows:

### 5.2. Screening under Controlled Conditions

#### 5.2.1. Greenhouse Screening

Conducting screening under controlled conditions using a greenhouse can be a useful technique to verify the outcomes of evaluating wilt-diseased plots (WDP). This is crucial for researching the molecular mapping and tagging of a specific disease race, as well as the inheritance of pathogens [85]. Furthermore, pathogenic diversity studies can be carried out under controlled circumstances to learn the disease’s genotypic information [89]. To screen the chickpea germplasm in greenhouses, the pot culture method has been standardized [97]. Another method that is frequently used for growing chickpea is root dip inoculation under greenhouse screens [94]. The identification of ninety percent of wilt in susceptible lines is guaranteed using the pot screening technique, although soil compaction from repeated irrigation may impair the association between pot and field performance. The chickpea seedlings are raised in autoclaved soil, dipped in inoculum at the roots, and then, transplanted into pots containing autoclaved soil, and the disease incidence is then measured [97]. There are some limitations to the greenhouse screening method, as well. It is very difficult to maintain uniform density of the inoculums in each diseased plot. So, it is not possible to differentiate the wilted plants in to early, late, and resistant categories.

#### 5.2.2. Laboratory Screening

Laboratory screening methods include various technologies, such as polymerase chain reaction (PCR), loop-mediated isothermal amplification (LAMP), quantitative PCR (qPCR), etc., for the accurate detection of *FOC*. In chickpea, artificial screening methods have been created by ensuring uniform inoculum load at the same vegetative stage of each test plant. This method guarantees that all inoculated plants have a roughly equal chance of infection by injuring the roots prior to inoculation [98]. Using this method, 25 resistant genotypes and 21 asymptomatic genotypes were identified. The method was applied to 211 genotypes from a core collection that included more than 16,000 unique chickpea germplasm accessions [70]. It has been suggested that pollen bioassays be employed as a quick and effective screening method to distinguish between resistant, late wilting, and susceptible genotypes [99]. One of the poisons produced by the fungus, fusaric acid (FA), is used as a selection agent to examine the genotypes of chickpeas.

## 6. Management of Fusarium Wilt in Chickpea

Management techniques to treat the disease are always adopted after a thorough disease evaluation. The management of Fusarium wilt in chickpea cannot be fully accomplished using a single control measure [100]. Elimination of the pathogen, as well as a reduction in the quantity and/or effectiveness of the main inoculums, are necessary for disease management [101]. The ideal control measure for such a goal should include the efficient application of one or a combination of the following management strategies:

### 6.1. Utilization of Pathogen-Free Planting Material

Fusarium wilt can be spread by infected seeds and plant waste [102]. Using infected propagation material, the pathogen is transferred into productive areas or soils that are pathogen-free. Therefore, the significance of monitoring the health of the item through certification programs under quarantine legislation and phytosanitary inspection should be taken in to consideration. The right choice of planting site is aided using *F. oxysporum* spp.-free planting material in non-infested soils [102].

### 6.2. Chemical Control

Chemical control is one of the finest disease management strategies for diseases that are spread through soil. FW can be controlled using organic chemical methyl bromide, which is a very effective fumigant. This chemical was used by Animisha et al. [100] to control FW. In addition to this, some popular fumigants, including dazomet, chloropicrin, carbendazim, and 1,3-dichloropropene, were also employed to combat FW in pea and chickpea, respectively [101].

### 6.3. Biological Control

An integrated disease management strategy can easily include biological control and plant resistance as a cost-efficient and environmentally beneficial method of disease control [102]. An effective cure for chickpea wilt disease has been demonstrated using an arbuscular mycorrhizal consortium to control the biological processes of Fusarium wilt [103]. Numerous biocontrol agents have been used effectively and have led to a significant decrease in both pathogenic fungal growth in vitro and disease development in plants [104]. These bacteria and fungi include non-pathogenic and non-host Fusarium species [105]. The *Pseudomonas fluoresces* formulation treatment has increased chickpea production in the field and can be applied as a seed treatment to prevent chickpea wilt. Additionally, Fravel et al. [106] linked higher plant defensive responses to root colonization by the non-pathogenic strain of *Fusarium* spp. with disease reduction [107]. In a study, it was discovered that pre-treating chickpea seedlings with *Rhizobium* isolates before subjecting them to *FOC* increased the levels of total phenolics, constitutive is flavonoids, for mononetin, and biochanin [108]. The protection of chickpea against Fusarium wilt by non-pathogenic and non-host Fusarium species has been linked to the induction of the phytoalexins medicarpin and maackiain, as well as the related isoflavones formononetin and biochanin A [109].

### 6.4. Cultural Control

Fusarium wilt disease in numerous crops was successfully controlled using the soil solarization method [110]. The heat produced by solarization may not kill the pathogen, but it may weaken it, reducing its host’s sensitivity and increasing its susceptibility to assault by other soil microflora members [111]. The risk of disease in the following crop could be reduced by clearing away the debris from a field that has been afflicted by Fusarium wilt and igniting or burning it to destroy the *FOC* chlamydospores. Temperature has a big impact on chickpea’s ability to resist Fusarium wilt. When there is a rise in temperature of 2–3 °C, different races of *Fusarium oxysporum* f. sp. ciceris (*FOC*) become more vulnerable to pathogens [112].

According to an investigation by Orr and Nelson [113], the Fusarium wilt pathogen in chickpea can live in the soil for up to 6 years, and 3 years of crop rotation is ineffective in lowering the incidence of the disease. In a 1998 study in southern Spain, Navas-Cortes found that planting date had the greatest impact on epidemic development. Sowing chickpea crops later in the year, from early spring to early winter, can slow the spread of Fusarium wilt epidemics and boost chickpea seed production [112].

### 6.5. Use of Resistant Cultivars

The most practical and cost-effective technique for controlling Fusarium wilt is the use of resistant cultivars. However, several factors that affect disease resistance, such as genetic and pathogenic variability, the evolution of the pathogen, the availability of resistance sources, the co-infection of plants with other pathogens, genetics, and the penetrance of resistance (i.e., reduced expression as a result of the interaction between host genotype and inoculum load, temperature, and seedling age), etc., can seriously limit its use and effectiveness [112,113]. A crucial element of the integrated disease management (IDM) program is the use of resistant chickpea cultivars and additive or synergistic combinations of biotic, cultural, and chemical control strategies [112]. The use of resistant cultivars has been restricted because certain novel materials have undesirable agronomic characteristics. Furthermore, the effectiveness and widespread use of current resistant cultivars may be constrained by the considerable pathogenic diversity of *FOC* populations [114].

Recent years have seen significant challenges in achieving the desired yield of chickpea due to various factors. In most chickpea-growing regions, studying different stressors is important [90]. Future work should therefore concentrate on creating cultivars that are multi-stress-resilient. A thorough comprehension of significant pressures and the genetics of resistance ought to result in more methodical methods of resistance breeding. It is important to breed wild Cicer species for resistance because they have a lot of potential [99].

## 7. Advanced Breeding Techniques

**Figure 3:**
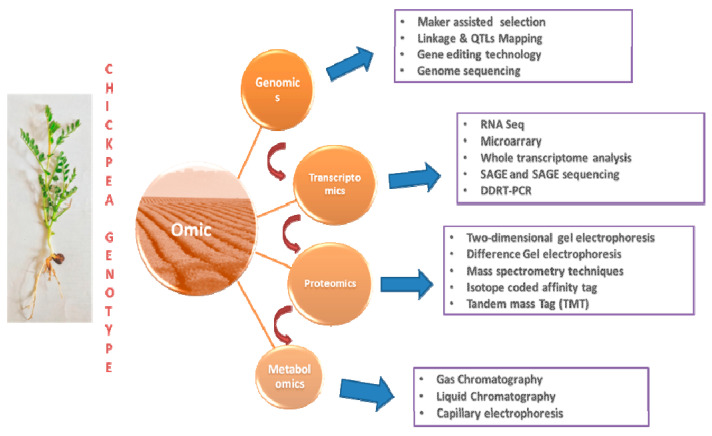
Omics approaches and their role in chickpea breeding.

The study of an organism’s entire genome is referred to as genomics. Recombinant DNA, DNA sequencing techniques, and bioinformatics are all combined in genomics to sequence, assemble, and analyze the structure and function of genomes [115]. Genomic science is the study of how genes and genetic data are structured inside the genome, the procedures for gathering and evaluating these data, and how this organization influences their biological usefulness. The three key fields of genomic biology are structural, comparative, and functional (Figure 3) genomics [116]. With the goal of understanding evolutionary linkages and how genes and genomes function to produce complex phenotypes, such as gene regulation and environmental signaling, genomics is a branch that aids in comprehending the sequencing of genes and genomes [117].

### 7.1. Marker Technology

There are three types of markers generally used in crop improvement programs including phenotypic, biochemical, and molecular markers [118]. Among these markers, molecular markers are more authentic due to their neutral behavior in different environmental conditions. Nucleotide sequences make up molecular markers, and the variation in nucleotide sequences among different individuals makes it possible to study these sequences [119,120]. The use of molecular markers that are closely related to the genes or QTLs controlling Fusarium wilt resistance allows for quicker and more accurate breeding. Although they are created through insertion, deletion, point mutations, duplication, and translocation, these polymorphisms are not always connected to the activity of the genes [121,122].

The genetic marker is a gene or DNA sequence with a known chromosome location that regulates a certain gene or characteristic. Genetic markers are closely related to the target gene and act as warning indications or flags [118]. Meanwhile, in contemporary genetics, genetic polymorphism describes the relative variation in the genetic loci of the genome. Genetic markers can be used to aid in the study of heredity and variation. Recent advances in molecular breeding, including the use of PCR-based techniques, such as simple sequence repeats (SSRs), insertion/deletion mutations (Indels), single-nucleotide repeats (SNPs), genomic sequencing (GS), genotype by sequencing (GBS), etc., have been widely used in crop improvement programs worldwide [119].

In contrast to multi-locus markers, including random amplified polymorphic DNA (RAPD), arbitrarily primed polymerase chain reaction (AP-PCR), inter-simple sequence repeat (ISSR), amplified fragment length polymorphism (AFLP), and sequence-specific amplification polymorphism (S-SAP) markers [120], the single-locus markers—including fragment length polymorphisms (RFLPs), variable number tandem repeats (VNTRs), simple sequence length polymorphisms (SSLPs), sequence-tagged microsatellite sites (STMSs), simple sequence repeats (SSRs), sequence tagged sites (STSs), single-nucleotide polymorphisms (SNPs), cleaved amplified polymorphic sequences (CAPSs) and sequence-characterized amplified regions (SCARs)—are frequently used in plant breeding in a variety of studies. In modern plant breeding, single-locus markers are used for various purposes, including germplasm characterization and protection, gene tagging, genome mapping, linkage map construction and analysis, evolution studies, parental selection, F_1_ hybrid testing, genetic purity testing of seeds, genes, QTL mapping, etc. [121,122]. Employing marker loci that are strongly connected to vital genes that regulate features with economic relevance, such as disease resistance, male sterility, self-incompatibility, and seed qualities (including form, size, color, and texture) can help in selection.

#### 7.1.1. Molecular Markers and FW Resistance in Chickpea

The identification and creation of genetic maps of the segregating population are breeder’s top priorities. Utilizing molecular markers for labeling traits and site-specific genes of interest, chickpea genetic maps have been created [123]. Using isozymes from the F_2_ population resulting from interspecific crosses, the first maps were produced [124]. Numerous studies have discovered genes that influence floral color, wilt resistance (Fusarium), double pods, and growth behavior [123,125]. Higher numbers of maps connected to features were derived using multiple markers, crosses from *C. reticulatum*, and other techniques. Microsatellite markers, however, were used to create populations from interspecific crosses, which take advantage of more genetic variations among chickpea genotypes [126]. The first transcriptome study of the chickpea genome was finished after the development of next-generation sequencing [127]. With the development of transcriptome information, detailed genetic maps were created using large-scale molecular markers [128,129,130]. The genetic population utilized to map and find QTLs in the chickpea genome may benefit from having access to draught genome sequencing in the Desi and Kabuli types [131]. Omics methods gathered genomic data and sparked the development of tightly connected QTLs in molecular markers [132].

The diseases for which significant resistance genes have been backcrossed into elite cultivars are the ones for which MAS in plant breeding is most effective [133]. Chickpea provides some evidence of the application of MAS to facilitate efficient and accurate breeding. The SSR markers, namely, TR19, TA194, and TA660, which were discovered to be polymorphic between the parental lines, have already been used for foreground selection via marker-assisted backcrossing in order to introduce *FOC*1 in a superior chickpea cultivar [134]. As part of marker-assisted introgression, the SSR markers TA110 and TA37 in chickpea LG2 were also used to introduce *FOC*-2 into the background of a superior cultivar [135]. To develop virtually isogenic lines with disease resistance, TA59, one of the several markers discovered to flank the *FOC* race 5 resistance gene, was used [136].

The use of molecular markers is an essential method for classifying, characterizing, and screening infections and diseases. To categorize and filter fungi, internal transcribed spacer (ITS) markers are often used. Even though information on pathogen variety is required to comprehend pathophysiology and development for management strategies, SSR markers are employed in unique backcross generation to aid in the selection against Fusarium resistance. The importance of resistant molecular markers in identifying disease-causing genes and resistance mechanisms has been acknowledged. Numerous crops have additionally demonstrated a substantial association between microsatellite markers and resistance genes, such as Fusarium wilt resistance genes, in chickpea, and many others.

Initial efforts to map resistance genes using restriction fragment length polymorphism (RFLP), RAPD markers, and isozymes failed. Only modest polymorphism was detected in chickpea using the resistant gene analogue (RGA), ISSR, and RAPD [137]. Nevertheless, *FOC*1 was mapped at 7.0 cm on the same side of the gene using two markers, viz., CS27700 and UBC170550. The resistance genes *FOC*3, *FOC*4, and *FOC*5 were later mapped using ISSR, RAPD, and SSR markers [138].

The first WR gene discovered was H_1_ against race 1 in chickpeas [139]. Two primers, UBC-170550 and CS-27700, respectively, amplified susceptibility and the DNA region linked to FW resistance [140]. However, after transforming these two markers into allele-specific associated primers (ASAPs), only CS-27700 was shown to be specific to the susceptible allele, whereas the other one (UBC-170550) appeared to be locus-specific. The same RAPD markers were later shown to be connected to the gene controlling race 4 resistance at 9 cm [141,142]. ISSR markers were also applied to tag the WR gene in a population that was inter-specific to the mapping method. The authors discovered two ISSR markers associated with the resistance gene for race 4: UBC-855500 and UBC-8251200.

SSR markers are the preferred markers for plant breeding or for plant breeders owing to their multi-allelic and co-dominant properties [143]. The development of SSR markers has made the application of genomic and transcript databases feasible. Several hundred SSR markers have been developed from genomic DNA libraries [144]. The “ICRISAT Chickpea Microsatellite” (ICCM) markers are a set of 311 distinct SSR markers that were created by Nayak et al. [144] using information from an SSR-enriched genomic library of the chickpea accession ICC 4958. Additionally, SSR markers (ESTs) have been mined using expressed sequence tags [144,145]. Primer pairs were created by Varshney et al. [145] for 177 unique EST-SSR markers, and 3728 SSR markers were found.

Using DNA markers, marker-assisted selection can expedite conventional breeding [146,147]. The resistant genotypes of chickpea that were discovered in this investigation may be employed in breeding programs to breed resistant cultivars. Previously, resistance to *FOC* races 1, 2, and 3 was delivered through genes 3, 2, and 1, respectively. The marker CS27 was first associated with *FOC* 1 at 7.0 cm by Mayer et al. [139], and later, this marker was modified to become an allele-specific related marker (CS27A). The *FOC*2 resistance gene was found at 2.7 cm and 0.2 from the SSR markers H3A12 and TA96. The formerly discovered DNA markers proved useful in establishing relationships to phenotypic data and connections to *FOC* 2 resistance genes. This was accomplished by using molecular markers, such as the ASAP marker (CS27700) and several STMS markers [139,148,149]. Utilizing the primers TA110, TR19, TS82, and CS27, a total of 28 genotypes were screened, and it was found that these genotypes were strongly related with *FOC* 2 resistance genes [138,150]. Resistance gene analogue, DNA amplification, fingerprinting, and other later-developed chickpea markers demonstrated more polymorphism compared to isozymes, RAPDs, and RFLPs. Nevertheless, the development of polymorphic markers led to substantial advancement in the discovery of STMS markers [151].

#### 7.1.2. Marker-Assisted Breeding

Marker-assisted selection (MAS), among other genomic methods, can significantly improve chickpea breeding programs [152]. How well MAS performs depends on the degree of association between the marker and the gene locus determining the target feature. The positioning of the marker in a genomic area with higher levels of polymorphism and simplicity of interpretation can affect the MAS technique [153]. The main advantage of MAS over traditional selection is the capacity to choose features that are difficult or inconvenient to assess directly, eliminating complicated and time-consuming evaluations. This is true when breeding for disease resistance is performed. By pyramiding different resistance genes in a single genotype, MAS also enables quicker variety release and development [154]. An effective technique for utilizing the potential of genes for agronomic traits is marker-assisted selection [155].

For orphan pulse crops, the success of MAS in cereal crops serves as a model. Many genetic resources have recently been invented and employed in marker-trait association research in pulses [156]. Under the auspices of the Indo-US Agricultural Knowledge Initiative (AKI) program, the Government of India, and the Indian Council of Agricultural Research (ICAR) launched the chickpea genomics initiative program.

##### Variations in MAS

The numerous molecular methods used in MAS include marker-assisted backcrossing (MABC), gene pyramiding, marker-assisted recurrent selection (MARS), and genomic selection (GS). In order to characterize genetic material and select individuals in the early segregating generation, these techniques have been applied in plant breeding, speeding up and improving the precision of the breeding cycle [157,158,159]. The genomics-assisted breeding (GAB) techniques MABC, MARS, and GS have recently been applied to breeding superior chickpea varieties with increased yield and resistance/tolerance to adverse climatic conditions [160].

##### Marker-Assisted Backcrossing (MABC)

MABC, a backcrossing technique, is made possible by molecular markers [161]. It expedites both the selection process and the genetic recovery of the recipient parents. By transferring the gene of choice or quantitative trait loci (QTLs) from the donor parent, this method is frequently used to eradicate undesirable features, such as disease and pest susceptibility, anti-nutritional factor, etc. from high-yielding cultivated varieties [162]. Foreground selection, background selection, and recombinant selection are the three steps of MABC.

Two high-yielding Desi cultivars viz., Annigeri 1 and JG 74, were employed in a collaborative effort between the University of Agricultural Sciences (UAS-Raichur) and Jawaharlal Nehru Krishi Vishwa Vidyalaya (JNKVV), Jabalpur, India, to increase FW resistance using the MABC method. In Central and South India, both grown species demonstrated high susceptibility to Fusarium wilt race 4 (*FOC* 4) and decreased production. This led to the development of two novel resistant varieties, namely, “Super Annigeri 1” and “enhanced JG 74”, by introgressing a genomic region that imparts resistance to *FOC* 4, utilizing MABC and WR 315 as the donor parent [163].

The two primary factors limiting the output of chickpeas are Ascochyta blight (AB) and Fusarium wilt (FW). Using a step-by-step MABC strategy, a superior chickpea cultivar, C 214, was given dual resistance [164]. The *FOC* 1 gene for FW and two quantitative trait loci (QTL) regions, ABQTL-I and ABQTL-II, were targeted for introgression to produce resistant lines. Employing foreground selection with six markers related to *FOC*1 and eight markers linked to both QTLs, it is now possible to choose plants with desirable alleles in several segregating generations. To find a plant with high recurrent parent genome recovery, background selection employing 40 uniformly distributed SSR markers was performed, in addition to foreground selection. After three backcrosses and three rounds of selfing, 22 BC_3_F_4_ lines for FW and 14 MABC lines for AB were acquired [165]. Three resistant lines for FW and seven resistant lines for AB have been identified phenotypically using this line.

##### Marker-Assisted Gene Pyramiding (MAGP)

One of the contemporary MAS methods used to produce MAGPs is the pyramiding of different genes. Two or more genes are picked for pyramiding simultaneously in MAGP. Gene pyramiding has been performed using an array of methods, including backcrossing, recurrent selection, complicated crossing, and multiple-parent crossing [166].

##### Marker-Assisted Recurrent Selection (MARS)

Recurrent selection, where two genes are chosen at a time for pyramiding, is an effective method used in plant breeding to improve quantitative traits through continuous crossing and selection processes [167]. The breeding cycle is slowed down by environmental changes, which have a negative effect on the breeder’s ability to select. At each generational level, molecular markers are employed for the intended features in MARS. Every cycle of crossing and selection in this case involves selectively crossing specific plants. The selection is made utilizing phenotypic data and marker scores. As a result, it accelerates the breeding or selection cycle and boosts the efficiency of recurrent selection. MARS is a forward breeding approach that has been extensively used for polygenic traits such as agricultural production and resistance to different biotic and abiotic stresses [168].

### 7.2. Genetic Mapping and QTL Technique

Studying the genetics of quantitative traits is crucial in the field of plant biotechnology. Complex quantitative features can be found in many plant species in nature. We now have better knowledge of these complicated traits. The section of the genome known as a QTL is linked to a quantitative trait’s influence [169]. Quantitative trait loci are made up of a single gene or a group of linked genes that affect phenotypes. One or more genes that influence quantitative traits have been identified using molecular markers and advanced statistical methods, together with specific chromosome loci. These identified loci are known as QTLs. For the attributes of yield, quality, insect-pest resistance, abiotic stress tolerance, and environmental adaptation in chickpea, QTL mapping is conducted. When identifying connected QTLs in a population with segregating traits, it is essential to select parents with a variety of genetic backgrounds and to hybridize parental lines that differ in one or more of their quantitative traits [170].

Genetic maps are created by employing the segregation and recombination principles of Mendelian genetics. They may demonstrate how close together chromosomes and DNA producers are within an organism. This level of parental differentiation in the population is crucial for the creation of genetic maps. Crop breeding and genetic mapping are closely related, and many crop breeding populations have already undergone genetic mapping [171]. Building genetic maps based on molecular markers that are easy to produce, highly repeatable, co-dominant, and specific to recognized linkage groups is greatly desired for breeding purposes. Because the length of each marker is the most crucial component, maps created using AFLPs, RAPDs, and ISSRs have limited transferability between populations and pedigrees within a species [171,172].

The identification and mapping of genes that impact chickpea resistance to different races of *FOC* have been made easier thanks to the use of DNA marker technology. In two mapping populations, CA 2156-JG 62 and CA 2139-JG 62, Halila et al. [172] discovered a second gene, *FOC*02/*FOC*02, which is flanked by markers TS47 and TA59 on LG2. Jendoubi et al. [173] used nearly isogenic lines (NILs) to finely map the *FOC*01/*FOC*01 gene on LG5 within a 2 cM interval. An SSR-based QTL analysis of the F_2:3_ population (C 214 × WR 315) identified two QTLs on LG6 for *FOC*1 resistance: FW-Q-APR-6-1 and FW-Q-APR-6-2 [174].

The first genetic maps of the chickpea were created using isozymes from F_2_ populations resulting from interspecific crosses. Following this, additional maps were created by various study groups. One of these maps included QTLs related to flowering time, agronomic traits, and Ascochyta blight resistance [175]. Other characteristics included double pod, growth habit, and Fusarium wilt resistance [176]. To map the *FOC*-3 resistance gene and connect it to the *FOC*-1, *FOC*-3, and *FOC*-4 resistance genes, RAPD, STS, ISSR, and STMS markers were used. At 0.6 cm from the *FOC*-3 gene, the STMS marker TA96 was found, but the STMS markers TA27 and CS27A co-segregated with TA96. Additionally, the authors found a link between *FOC*-3, *FOC*-1, and *FOC*-4. While *FOC*-1 and *FOC*-4 were mapped close together at 1.1 cm, *FOC*-3 appeared to be associated with them at distances of 9.8 cm and 8.7 cm, respectively.

Using the SSR marker TA103, *FOC*1 was introduced from WR 315 to C 214. Earlier, scientists discovered *FOC*1 flanked by the SSRs TA110 and H3A12 on LG2. On LG2, the genes for *FOC*2 (TA96-H3A12) and *FOC*3 (TA194-H1B06y) were also discovered. However, according to Jingade and Ravikumar [177], a major QTL (GSSR 18-TC14801) on LG1 for *FOC*1 resistance can account for up to 71% of phenotypic variance (PV). Moreover, a sizable QTL (FW-Q-APR-2-1) was found on CaLG02, and two smaller QTLs (FW-QAPR-4-1 and FW-Q-APR-6-1, respectively) were found on CaLG4 and CaLG6, indicating resistance to *FOC*1 and *FOC*3 [178]. It has been determined that the resistance loci on LG2 are either monogenic or oligogenic with respect to *FOC* 5. With the help of SNP and SSR markers, the possible LG2 genomic area was recently reduced to 820 kb [179].

Moreover, two distinct genes that provide race 0 resistance have been identified and labeled. The first resistance gene, *FOC01*/*FOC01*, was flanked by two markers, i.e., OPJ20600 and TR59, on linkage group 3 (LG3), which corresponds to LG2. In an F_2:3_ mapping population of “C 214” × “WR 315”, Sabbavarapu et al. [174] recently revealed two unique QTLs for race 1A (*FW-Q-APR6-1* and *FW-Q-APR-6-2*). The second gene (*FOC02*/*FOC02*) was located on LG2, and the STMS markers TS47 and TA59 were located on each side of it (Table 4). All additional wilt pathogen resistance genes were found in linkage group 2, except for *FOC*-01 and two QTLs for race 1A.

Numerous studies have shown that four genes, including *FOC*-1, *FOC*-3, *FOC*-4, and *FOC*-5, should be in the same linkage group [180]. Five resistance genes, viz., *FOC*-1, *FOC*-2, *FOC*-3, *FOC*-4, and *FOC*-5, were found to be clustered in chickpea. On LG2, a cluster of five genes covering 8.2 cm was discovered. The resistance gene cluster was 2.952 Mb in size, where 1 cm equals 360 kb. Among the five genes, *FOC*-1 and *FOC*-5 were separated by 2.0 cm, but *FOC*-5 was separated from *FOC-3* by 3.4 cm. It was determined that 5.4 cm separated *FOC*-1 from *FOC*-3. There was a 1.0 cm distance between *FOC*-3 and *FOC*-2 and a 1.8 cm distance between *FOC*-2 and *FOC*-4. At the extremities of the cluster, 8.2 cm separated two genes (*FOC*-1and *FOC*-4). It was observed that gene order and map distances were more accurate because only one source of resistance to five genes was utilized, and the mapping population descended from an intraspecific cross.

The discovered QTLs for various traits can be utilized in genomics-assisted breeding using modern techniques, such as marker-assisted backcrossing, the introgression of superior alleles from wild species through advanced backcross QTL, marker-assisted recurrent selection, and genome-wide selection. Garg et al. [178] constructed a genetic map for resistance to Fusarium wilt on 188RILs gene rated from a cross between JG 62 and ICCV 05530, and identified five QTLs for resistance, with explained phenotypic variance ranging from 6.63 to 31.55 percent. Out of the five QTLs found, three QTLs onCaLG02 and one minor QTL each on CaLG04 and CaLG06 were mapped for race1.

According to molecular mapping investigations, resistance genes for pathogen races 0, 1, 2, 3, and 4 have been found on LG2 of the chickpea map. Due to the grouping of six resistance genes, LG/2 is a hotspot for Fusarium wilt resistance. In order to employ MAS and better understand the molecular mechanism of resistance, strongly related markers for some of the genes have been found and verified in various genetic backgrounds [181]. Race 5 resistance gene near-isogenic lines have been created, which can be used for map-based cloning and fine mapping.

*FW-Q-APR-2-1*, a significant QTL for race 1, was identified on CaLG02. Additionally, minor QTLs on CaLG04 (*FW-Q-APR4-1*) and CaLG06 were detected (*FW-Q-APR-6-1*). For race 3 of an FW discovered in Ludhiana, a significant QTL was discovered on CaLG02 (*FW-Q-APR-2-1*) and CaLG04 (*FW-Q-APR4-1*). Since the primary QTLs for races 1 and 3 on CaLG02 shared flanking markers, i.e., TR19 and H2B061, it is possible that the same genomic regions regulate resistance to these two races.

### 7.3. Genome Sequencing

A few decades after the Sanger DNA sequencing method was created, deep, high-throughput, in-parallel DNA sequencing techniques known as next-generation sequencing (NGS) were created. Amplification libraries, also known as amplified sequencing libraries, are required for second-generation sequencing methods. It is now possible to perform single-molecular sequencing by employing third-generation sequencing, without the time-consuming and expensive amplification libraries. Research teams may now create de novo draught genome sequences for every organism of interest, with the help of bioinformatics tools and the synchronized rapid advancement of NGS technology. These technologies can be applied to whole-transcriptome shotgun sequencing (WTSS, also known as RNA sequencing (RNA-seq)) [182], targeted (TS) or candidate gene sequencing (CGS) [183,184], whole-exome sequencing (WES) [185], and methylation sequencing (MeS) [186].

Genome sequencing is being transformed due to advances inhigh-throughput technology. The intense rivalry among new sequencing techniques has led to some incredible advancement. The essential concepts of the best-known sequencing platforms are: ABI/SOLiD sequencing, Roche/454 Life Sciences sequencing, and Solexa/Illumina sequencing.

Prior to 2013, the chickpea was recognized as an orphan crop due to a lack of genetic data. However, the first draughts of the genomes of the Desi and Kabuli chickpea investigations were released in 2013 [187]. The development of high-throughput sequencing and next-generation technologies laid the foundation for the sequencing of the chickpea genome. A thorough map of deviation in 3171 cultivated and 195 wild accessions was produced by Varshney et al. [188] to provide resources for breeding and research on chickpea genomics.

The creation of genetic resources is still crucial for molecular or genomics-assisted breeding. Unfortunately, there has been delayed development of genetic resources for this important crop of legumes. Chickpea genomic resources have significantly increased in recent years due to next-generation sequencing (NGS) initiatives and their use in genomics research [188]. The discovery of the candidate gene(s)/genomic regions controlling disease resistance may be made possible by the availability of whole-genome sequence information in different plant species, including chickpea. Williams et al. [189] and Srivastava et al. [190] reported on the virulence-related genes *FOC* (*FOC*-38-1) and Fop (Fop-37622), which have provided fresh information that has increased our comprehension of the pathogenicity of FW and the evolution of the host–pathogen interaction in legume species.

The use of NGS technology has led to the creation of numerous molecular markers for the advancement of chickpeas [188]. In the past, millions of SNP markers, 2000 SSR markers, and more than 15,000 feature-based diversity array technology (DArT) platform markers have been produced for chickpea. The NGS revolution has made it possible to perform sequencing at different depths, including whole-genome re-sequencing, skim sequencing, and low-depth sequencing (genotyping via sequencing, RAD-Seq).

## 8. Multi-Omics Approaches

Several interesting omics technologies have evolved during the past few decades. The information gathered using these omics techniques may be combined with genetic information to alter a variety of biological processes involved in chickpea breeding. These omics-based techniques have been proven to be useful for examining the molecular and genetic foundations of crop development by modifying DNA, proteins, metabolites, transcript levels, and mineral nutrients against negative environmental and physiological stress responses [191]. Numerous omics methods have disclosed each corresponding molecular biological aspect integrated with plant systems, including metagenomics, genomics, transcriptomics, metabolomics, proteomics, ionomics, and phenomics [192]. High-throughput and speedy data creation for transcriptomes, genomes, proteomes, metabolomes, epigenomes and phenomes has been made possible by the development of next-generation sequencing (NGS) technology [193]. The integration of different omics techniques under physiological and environmental stress could reveal gene networks and activities [15]. The use of omics provides a systems biology approach to comprehending the intricate relationships between genes, proteins, and metabolites within the phenotype. In order to preserve and develop crops, this integrated approach largely relies on computational analysis, bioinformatics, chemical analytical procedures, and many different biological disciplines [194]. For the purpose of finding possible candidate genes and their pathways, the integration of various omics methods may prove useful. Omics allows for a deeper understanding of the processes behind the complex architecture of numerous phenotypic features with agricultural importance. Thus, the integration of multi-omics approaches may be beneficial to identify the mechanisms behind the expression of simple and quantitative traits such as higher yield and disease resistance. Omics approaches are also important for understanding the inheritance of these traits [195]. This information is significant in the development of biotic stress-resistant cultivars through the introgression of desired traits to maintain the sustainable production of different crops, including chickpea. For example, metabolomics may help in the identification of the up-and down-regulation of different metabolites that are important for defense systems in plants [196].

### 8.1. Transcriptomics/Gene Expression Studies

Differential gene expression in chickpea plants infected with Fusarium wilt, as well as plants without infection, comparatively offers a wealth of resources for the functional analysis of resistance-related genes and their application in breeding for long-lasting wilt resistance. In chickpea, various studies have been conducted to identify differentially expressed genes. Using cDNA-RAPD and cDNA-AFLP techniques, Nimbalkar et al. [182] identified differentially expressed genes in chickpea during root infection by *Fusarium oxysporum* f. sp. ciceri race 1. Based on a cDNA template and decamer primers, the former discovered nine transcripts that were differently expressed in the infection-resistant chickpea variety. In total, 273 of the 2000 transcript-derived fragments (TDFs) displayed differential expression in infected chickpea stems. Only 13.65% of the TDFs were differentially expressed during the pathogen infection process in chickpea roots, while the remaining 86% did not vary in expression (Table 5). In a study, Saable et al. [196] identified 162 DEGs that belonged to defense signaling pathways. Using this sequence, other studies have also been carried out to discover differentially expressed genes (DEGs). Ashraf et al. [197] discovered 6272 DEGs that belonged to stress-responsive genes in chickpea through RNA blot analysis during wilt infection with race 1. Gupta et al. [198] race 1 induced redox state alterations in chickpea. Recently, Priyardashni et al. [199] analyzed the expression of NBS-LRR and WRKY genes in chickpea infected with Fusarium wilt, causing a fungal pathogen.

The transcriptome, or the complete collection of RNA transcripts produced by an organism’s genome in a cell or tissue, is the subject of the study of transcriptomics [208]. To study how genes are expressed in response to various stimuli over an extended period, a dynamic technique called transcriptome profiling has grown in popularity [209,210]. By enabling the researcher to examine the differential expression of genes in vitro, this method aids in the clarification of a gene’s basic function. To analyze transcriptome dynamics, at first, conventional profiling approaches, such as differential display-PCR (DD-PCR), SSH, and cDNAs-AFLP, were used; however, these methods had poor resolution [211]. The use of microarrays, digital gene expression profiling, NGS, RNA seq, and SAGE for RNA expression profiling was soon made possible through the development of truthful techniques [212,213]. A breakthrough technique for advancing transcriptomics uses in situ RNA-seq, often referred to as in situ ligation, to sequence RNA in living cells or tissues [214]. A second method called spatially resolved transcriptomics uses spatial information to detect gene expression within cells or tissues in order to provide a detailed molecular description of physiological processes in living things [215]. One of the better techniques for creating genic-SSR markers that can be connected to phenotypic features associated with candidate genes is RNA-seq.

Before the discovery of digital transcriptome profiling, expressed sequence tags (ESTs), cDNA-AFLP, and cDNA-RAPD were mostly employed to identify the gene(s) involved in plant defense mechanisms and plant–pathogen interactions [216,217,218,219,220]. In recent years, the transcriptome analysis of the four chickpea cultivars, viz., JG 62, ICCV 2, K 850, and WR 315, allowed the genomic regions regulating FW resistance to have “big effect” SNPs and Indels [221,222]. The chickpea from the cross ILC 3279 × WR 315 was functionally validated for the genomic area determining *FOC* (race 5) resistance [223]. In this experiment, resistant and sensitive NILs were generated. Three novel candidate genes, i.e., LOC101495941, LOC101509359, and LOC101510206 (encoding the MATE family protein, MADS-box transcription factor, and serine hydroxymethyl-transferase, respectively) and two previously known candidate genes, i.e., LOC101490851 and LOC101499873 (encoding chaperonin) were related to defense activity against FW, recognized via differential gene expression analysis at twenty-four hours post inoculation (hpi) [224]. Numerous transcripts associated with distinct TFs were found to be differently expressed in JG 62 and WR 315 in response to FW (race 1) infection. Through sugar metabolism and cellular transporters, defense signaling against FW was activated in chickpea [224].

### 8.2. Proteomics and Metabolomics

Proteomics is a method used to profile all the proteins that are expressed in an organism. It is broken down into four separate categories: sequence, functional, structural, and expression proteomics [225,226]. Traditional proteomics includes size exclusion chromatography (SEC), exchange chromatography (IEC), and affinity chromatography. Western blotting and an enzyme-linked immune sorbent assay can be utilized to analyze specific proteins (ELISA). Additionally, more advanced methods for the separation of proteins have been developed and employed, including SDS-PAGE, 2-DE, and 2-D differential gel electrophoresis (2D-DIGE).

The numerous proteins involved in host–pathogen interaction and their function in protecting the host plant from pathogen attacks can be uncovered using a proteomics method [227,228]. Many proteins have been linked to significant host–pathogen interactions, including the establishment of the pathogen in a host plant that is vulnerable to it, as well as the host plant’s defense against pathogen invasion [229,230,231,232]. These proteins range from syntaxins to subtilin-like proteases in different plant species in response to FW infection. They include chitinases, -1,3-glucanases, xylem proteinases, proteinase inhibitors, leucine-rich repeat proteins, proline-rich glycolproteins, pathogenesis-related (PR) proteins, cellulose synthases, ankyrin repeat-containing protein, and PR-5b [228,233,234,235].

The genotypes JG 62 (FW-susceptible) and Digvijay (FW-resistant) of chickpea were both found to contain a variety of defense-related proteins against FW infection [228]. Several ROS-activating enzymes, including glutaredoxin, glutathione peroxidase, ascorbate peroxidase, glutathione S-transferase, and peroxiredoxin, were identified in higher concentrations in Digvijay than in JG 62. This is similar to how Digvijay was able to reduce FW pathogen assault compared to the FW-sensitive cultivar JG 62 due to the genotype’s excess of PR proteins [228]. Proteomics may therefore improve our understanding of the unknown proteins linked to numerous signal transduction pathways that cause host innate immunity in grain legumes to be triggered in response to FW attack.

Metabolomics is the complete study of metabolites that participate in many cellular processes in a biological system. The total collection of metabolites generated by metabolic pathways in the plant system is referred to as the “metabolome”, instead [236,237]. The early metabolic system of an organism can be employed to predict its genome sequencing using metabolomics and NGS technology [238]. In one study, information was combined using the genome sequencing method (NGS) and metabolite measurement method (MS) to generate crop enhancement methods [239]. This can improve our understanding of how plants respond metabolically to stress via contact with pathogens or under stress.

Our understanding of many metabolites, hormonal interactions, and signaling components associated with plant defense systems against FW infection in agricultural plants, including grain legumes, may facilitate the development of resistant cultivars [228]. Hexokinase, trehalose, invertase, sucrose synthase, -amylase, and glucose-6-phosphate are examples of sugars that are generated in the reaction to FW [240]. These sugars act as an oxidative burst substrate, supplying energy, generating ROS, acting as a signaling molecule in coordinate on with various phytohormones, and enhancing lignification of the cell wall in order to activate plant innate immunity, and plays a crucial role in plant defense against pathogen attacks [241,242]. There are many different proteins that are involved in the TCA and glycolysis processes in Digvijay, as well as defense-related metabolites such as endo beta-1,3-glucanase, caffeic acid O-methyltransferase, chitinases, and caffeoylCoA O-methyltransferase; phytoalexins such as luteolin, genistein, and quinone; and phenolic compounds, including flavonoids [228]. A considerable decrease in specific amino acids and carbohydrates, like sucrose and fructose, in a vulnerable crop enables FW pathogens to enter and hasten the development of disease [228].

The function of PR proteins, chitinases, ROS activating enzymes, flavonoids, phenolic compounds, and phytoalexins in conferring wilt resistance is further supported by thorough analyses of plant transcriptomes, metabolomes, and proteomes in response to FW disease [243,244,245].

## 9. Genomic Selection (GS)

A promising method called genomic selection (GS) uses molecular genetic markers to create new breeding programs and new marker-based models for genetic valuation [246]. It offers chances to boost the genetic gain of complex traits per unit of effort and expense in plant breeding. For GS, weighing the pros and cons of working in crop plants is crucial. The most crucial elements for its successful and efficient application in crop species are the availability of genome-wide high-throughput, affordable, and flexible markers, and its low as certain bias, suitable for large population sizes, as well as for both model and non-model crop species with or without the reference genome sequence [247]. However, in order to achieve evaluable genetic gain from complex traits, these marker technologies may be paired with high-throughput phenotyping.

Most of the molecular markers, which have both large and small marker effects, are what determine the GS. Molecular markers are chosen based on their total genome coverage, and all QTLs should be in linkage disequilibrium with at least one marker [248]. The training population and the testing population are two separate sorts of populations that are employed in GS. The testing population, which is related to the breeding population, is used to estimate the genomic selection model parameter. A testing population is a population group in which genetic selection is employed.

One important issue with marker-assisted selection is that it can only target significant QTLs or genes. It is now commonly acknowledged that a multitude of genomic regions, each of which has just a tiny amount of genetic control, are involved in many complex traits, such as yield or broad-spectrum disease resistance. In many situations, it is highly advantageous to select for all or a few QTLs linked to the desired characteristic [249]. In this case, genomic selection, which has the capacity to capture several genes with minor additive effects, could prove beneficial for crop breeding. Genomic prediction, which relies heavily on the availability of high-throughput genotyping, along with accurate phenotyping data, is the key to success in GS breeding [90]. GS + de novo GWAS and haplotype-based GS + de novo GWAS approaches, together, have potential for developing capable chickpea genotype(s) [90].

## 10. In Vitro Selection against Fusarium Wilt Disease Tolerance/Resistance in Chickpea

Both biotic and abiotic stressors have a significant impact on legume crops. Therefore, it is essential to undertake efforts to cultivate plants that are tolerant to stress in order to increase agricultural yield. Growing stress-tolerant plants using tissue culture-based in vitro selection has become a practical and economical approach in recent years [250,251,252]. Applying selective agents to the culture media, such as pathogen culture filtrate [253], fusaric acid phytotoxin [254] or the pathogen itself (for disease resistance)—NaCl (for salt tolerance), and PEG [255] or mannitol for drought tolerance—may aid in the development of plant tolerance to both biotic and abiotic factors. Many efforts have been made in this respect for the screening and development of chickpea cultivars [256].

The optimal outcome depends on the availability of an appropriate selection agent. Fungal culture filtrate or a well-known toxin, such as oxalate acid or fusaric acid, are typically utilized as the selection agents [257]. In vitro pathogen resistance selection is possible by including a phytotoxin, such as fusaric acid, that is unique to the host. Fusaric acid (C_10_H_13_O_2_N), a metabolite generated by many strains of *Fusarium oxysporum*, is employed as a “selecting agent” for cell culture and callus culture to stop the germination of fungus. In comparison to plants derived from tissue culture without selection, several pathogen-produced non-specific phytotoxins, such as deoxynivalenol (DON), crude pathogen culture filtrate, or sometimes, the pathogen itself, have been shown to increase the frequency of resistant/tolerant plants [258]. Because there is a link between toxin tolerance and disease tolerance, toxin or filtrate can be used to make an agent decision based on reality. By exposing somatic embryos, shoots, embryogenic calli, or cell suspensions [259,260] to pathogen toxins, pathogen culture filtrate, or the pathogen itself, these selections can be made.

*Fusarium oxysporum* cultural filtrate affected the levels of total peroxidase, phenol, and beta 1, 3 glucanase in chickpea and reduced callus growth [261]. Resistance was apparent in chickpea plants that had grown back after being exposed to culture filtrate (*Fusarium oxysporum*) [262]. According to research conducted by Hamid and Strange [257] on the relationship between disease and the susceptibility of chickpea shoots to toxins (Solanapyrone A, B, and C) and the culture filtrate of *Fusarium oxysporum* (*Ascochyta rabiei*), the enzyme glutathione s-transferase may prove useful for boosting resistance.

## 11. Speed Breeding in Chickpea Improvement

Crop varieties that are resistant to disease can be developed using plant breeding techniques [258,259]. In order to protect global food security, it is urgently necessary to increase the existing pace of genetic gain in key food crops [260,261]. This may be helpful in the fast transfer of desired genes [262]. Lengthy breeding cycles/generations are mostly to blame for the poor advances in crop improvement [263]. Traditional/conventional breeding methods may not be sufficient to meet the demands of future generations. Speed-breeding approaches are increasingly applied at large/small scales to obtain rapid genetic gain in several crop species in order to overcome the limitations associated with traditional methods and to ensure food security [264]. Crop varieties can be developed more quickly through speed breeding. This involves a synthetic habitat that has longer daylight hours to extend the growing season and aid in the manipulation of photo insensitive crop life cycles [265].

The rapid generation cycling methods of double haploids [266], the in vitro culturing of immature embryos [267], the embryo rescue technique [268], and other methods have not been successful in the chickpea. Three generations per year in short-season conditions were supported in the first report on chickpea rapid generation development [269]. It may be advantageous to increase production and reduce life cycles using the recently established “speed breeding” technique in chickpea, which could let researchers conduct more generations per year [270,271]. In the pigeon pea plant, a rapid generation advancement approach, which showed 100% germination from immature seeds taken from 35-day-old plants, opened new possibilities for developing three to four generations in a year [272].

The induction of early blooming and the germination of immature seeds in a more recent study on chickpeas resulted in a shorter seed-to-seed cycle period [273]. A system for growing chickpeas in greenhouses with artificial light but no growth regulator has been developed. In this technique, the photoperiod must be extended to 22 h using a temperature-controlled greenhouse with working high-pressure lamps. This greenhouse provides for precise control of temperature, humidity, and lighting. Immature seeds were germinated at 20–23 days after flowering (DAF) to further shorten the generation cycle, and the photoperiod was increased to encourage early flowering. There were six accessions used, with two each from the early, medium, and late maturity groups. Six or seven generations occurred annually. This method may also be adopted for the screening of wilt-resistant plants, as it may save time.

According to Fikre and Tulu [274], a unique field-based rapid generation cycle strategy may increase breeding effectiveness and hasten the release of improved chickpea varieties for the farming community. Compared to other rapid generation progress technologies that require expensive infrastructure, the strategy is easy to use, effective, and requires little investment. Importantly, the field-based rapid cycle technique for chickpeas is best suited for breeding operations located in tropical and subtropical areas, where the climate allows for chickpea development all year round. However, because this strategy includes propagating plant generations outside, it is crucial to implement risk management procedures to safe guard priceless breeding resources from severe weather conditions and wildlife. Speed breeding strategies may also be applied to the development of Fusarium wilt-resistant chickpea varieties.

## 12. Conclusions

Biotic stressors significantly decreased the yield of the leguminous crop. After yield improvement, resistance to FW one of the most important breeding goals of crop improvement programs for chickpea. The development of efficient, innovative, conventional, and molecular breeding technologies can be used to strategically control breeding for FW resistance. This review has covered the many approaches that may be utilized to locate and incorporate novel wilt resistance gene in chickpea. The capacity to apply a QTL mapping strategy for the genetic study of stressors in chickpeas was made possible by recent advancements in the utilization of molecular marker technologies and the availability of high-density genetic maps. Draft chickpea genome sequences have since been made public. Due to the significantly increased chickpea genomic repertoire, we have a fantastic opportunity to examine the unique genetic determinants/haplotypes governing this stress across the full genome level using genome-wide association studies (GWAS). Several marker-assisted breeding methods, including MABC and MARS, are now being applied in chickpea breeding programs. To understand functional analyses, the molecular mechanisms of genes, and gene networks, these omics approaches—genomics, transcriptomics, proteomics, metabolomics, ionomics, and phenomics—have been employed. There is an urgent need for the identification of transcription factors that play an important role in limiting the pathogen activity of *Fusarium oxysporum* in the soil, as well as in chickpea. This review outlines approaches that can be used to manage the effect of FW on chickpea production and suggests recommendations for improving chickpea wilt-resistant breeding programs. The adoption of these approaches is anticipated to be given more prominence in future breeding programs. This review includes information on the biotic limitations of chickpea production and future directions.

## Figures and Tables

**Table 2:** Important cultivars/donors (genetic resource) contributing to Fusarium wilt resistance.

Important Varieties/Donors	Country	Reference
Surutato-77, Sonora-80, UC-15, UC-27, and Gavilan	Mexico	[27]
BG-312, ICCVs 98505, 07105, 07111, 07305, 08113, and 93706, ICCVs 08123, 08125, 96858, 07118, 08124, 04514, 08323, and08117(moderately resistant)	India	[85]
WR 315, JG 315, CPS 1, JG 74, Avrodhi, and Phule G	India	[84]
ICCV 2,3,4,5 and ICC 11322, 14424, and 14433 (against race I)	India	[88]
Digvijay	India	[89]
ICC 14194, ICC 17109, and WR 315	India	[90]
Three lines derived from MABC-based C 214 and WR 315 cross	India	[91]
ICCV 09118, ICCV 09113, ICCV 09115, ICCV 09308, ICCV 09314, ICCV 05527, ICCV 05528, and ICCV 96818	India	[73]
Super Annigeri and improved JG74 (resistant against *FOC*4)	India	[92]
ICC 7537 resistant to all races (except race 4)	Ethiopia	[27]
FLIP 84-43C (against race 0), ILC-5411, FLIP 85-20C (against race 5), FLIP 85-29C, FLIP 85-30C, ILC-127 (against race 0), ILC-219 (against race 0), ILC-237, ILC-267, and ILC-513 (against race 0)	Santaella, Córdoba, Spain	[93]
Annigeri	India	[27]
ICC-7520	Iran	[27]
Andom1 and Ayala	-	[63]

**Table 3:** Details of scoring scale to calculate Fusarium wilt disease incidence in chickpea.

Rating	Wilt/Mortality (%)	Field Observation
1	0%	No lesions visible
2	<10%	Few scattered lesions, usually seen after careful examination
3	11–20%	Lesions and defoliation on some plants; little damage
4	21–50%	Lesions very common and damaging; 25% plants killed
5	51–80%	All plants with extensive lesions, causing defoliation and drying of branches; 50% plants killed
6	>81%	Lesions extensive on all plants; defoliation and drying of branches; more than 75% plants killed

**Table 4:** List of various QTLs contributing to Fusarium wilt in chickpea.

Fusarium Race	Name of Population	QTLs	Marker Identified	Linkage Group	References
Race 1 Race 4	C-104 × WR-315	-	CS-27700, UBC-170550 (RAPD)	-	[140]
Race 3	WR-315 × C-104	*FOC-3*	TA96 and TA27, TA196 (STMS)	-	[26]
Race 1 Race 4	-	*FOC-1* (syn. h (1)) and *FOC-4*	CS27A (STS/SCAR) TA194 (STMS)	-	[61,138]
Race 5	-	*FOC-5*	TA59 and TA96 (SSR)	-	[174]
Race 2	-	*FOC-2*	TA96 and H3A12 (STMS)	-	
Race 4 Race 5	*C. arietinum* × *C. reticulatum*	-	STM S and a SCAR	-	[138]
Race 1	F9	*FOC-1*	H3A12, TA110 (STMS)	-	[61]
Race 0	CA 2139 × JG 62	*FOC01*/*FOC01*	OPJ20(600) (RAPD) TR59 (STMS)	LG3	[138]
Race 0	CA 2139 × JG 62	*FOC02*/*FOC02*	TA59 (STMS)	LG2	[174]
Race 1A	C 214 × WR 315	*FW-Q-APR-6-1* (*FOC-1*) and *FW-Q-APR-6-2* (*FOC-1*)	CaM1402 and CaM1101 (flanking) CaM1125-TA22	LG6	[176]
Race 5	-	*FOC-5*	TA59 (STMS)	LG2	[59]
Race 1	JG 62 × WR 315	-	TA27-TA59 (STMS)	LG2	[4]
Race 1 Race 3	C 214 × WR 315	*FOC-1* and *FOC-3*	GA16, TA110, and TS82	LG2	[134]
Race 1	JG 62 × ICC V05530	3QTL (race 1), *FW-Q-APR-2-1* *FW-Q-APR-4-1* *FW-Q-APR-6-1*	TR19 and H2B061, TA132 and TA46 (STMS)	CaLG02, CaLG04, and CaLG06	[178]
Race 3	JG 62 × ICC V05530	2QTLs (race 3) *FW-Q-APR-2-1* and *FW-Q-APR-4-1*	CKAM1256 and TS72	CaLG02 and CaLG04	[178]
Race 0	CA 2156 × JG 62	*FOC01*/*FOC01*	H2I20 and TS43 (STMS)	LG5	[58]
Race 5	WR 315 × ILC 3279	*FOC-5*	TA59, CaGM07922, and SNPs	LG2	[179]
Race 4	Annigeri1 × WR-315	*FOC-4*	TA59, TA96, TR19, and TA27	LG2	[164]
Race 4	JG 74 × WR 315	*FOC-4*	GA16andTA96		[164]
Race 5	-	*FOC-5*/*FOC-5*	TA27 and TA59 TA96 CS27_700_ (RAPD) UBC170550 (RAPD)	LG2	[57,138]
Race 5	-	*FOC-5*/*FOC-5*	ECAMCTA07 OP-M20-21045 OP-M20-31103	LG2	[164]

**Table 5:** Differentially expressed genes (DEGs) contributing to FW resistance in chickpea.

Genotype Used in Study	Platform/Technology	Differentially Expressed Genes (DEGs)/Candidate Gene	Study Based on	References
WR 315 and JG 62	cDNA-RAPD and cDNA-AFLP	273 DEGs related to stress response, gamma-glutamyl-cysteine synthetase, and *NBS-LRR*	Race 1	[182]
	RNA blot analysis	6272 ESTs belonged to stress-responsive genes and cell signaling, transcription, RNA processing, modification, cellular transport, homeostasis, and hormone response-related genes	Race 1	[197]
	Suppression subtractive hybridization	162 ESTs belonged to genes responsible for defense signaling pathways, energy metabolism, cell rescue, and superoxide dismutase	Race 4	[196]
	qPCR, Microarray analysis	Stress-responsive and other defense-associated genes, including aquaporin, ATP synthase, immunity-associated genes, cystatin and DnaJ, pectinesterase and xyloglucosyl transferase, actin- and profilin-like genes, cytochrome P450, and peroxidase	Race 1	[197]
	qPCR	Transporter gene, transporter like gene, redox regulatory respiratory burst oxidase homolog F (RBOHF), thioredoxin 3 (TRX3), cationic peroxidase 3 (OCP3), flavodoxin-like quinone reductase 1 (FQR1), iron superoxide dismutase 1, NADH cytochrome b5 reductase (CBR), Fe (II) oxidoreductase 7 (FRO7), genes related to intracellular transportation ABC transporter-like gene, polyol transporter gene, translocase, heavy metal transporter (detoxifying protein) (FRS6), bZIP, homeodomain leucine zipper, MYB, helix loop helix, zinc finger (CCHC type), heat shock family protein, sucrose synthase (SUS4), b-amylase (BAM1), serine threonine kinase (CDKB1.1), and vacuolar ATPase (TUF)		[198]
	Expression analysis	NBS-LRR and WRKY genes		[199]
Digvijay and JG 62	qPCR	Stress-responsive genes		[200]
	qRT-PCR and LongSAGE	3816 DEGs and G protein b subunit gene lignification, hormonal homeostasis, plant defense signaling, ROS homeostasis, and R-gene mediated defense		[201,202]
	qRT-PCR	5 DEGs related to stress-responsive category, glycosyltransferase gene, *GroEs2*, *60srp*, and *Betvi E*	Races 1, 2, and 4	[203]
ICC4958	Illumina (NGS) and Poly(A)-based qRT-PCR	122 conserved miRNAs, 59 novel miRNAs, and defense gene encoding Toll/Interleukin-1 receptor–nucleotide binding site leucine-rich repeats miR2111 targets a Kelch repeat-containing F-box protein		[204]
NILs—RIP8-94-5/RIP8-94-11	qPCR	22 potential defense-related genes encoding a MADS-box transcription factor, and TMV resistance protein	Race 5	[205]
WR315 and BG256	Sequencing (Roche 454 GS FLX system)	202 DEGs related to polyubiquitin, chlorophyll a-b binding protein, ferredoxin-NADP, translation factor sui1, carbonic anhydrase, ribulose bisphosphate carboxylase, oxygen evolving enhancer, elongation factor 1-alpha, and post-translational modification genes		[206]
JG 62, WR 315 and JAKI9218	qRT-PCR	6 DEGs, including transcription factors such as extracellular calcium-sensing receptor, Nitric oxide reductase, growth hormone-releasing hormone receptor, Cytochrome C oxidase Cbb-3 type subunit I, Hydroxynitrite lyase, Tir chaperone, and ionotropic glutamate receptor	Races 2 and 4	[207]

## Data Availability

Not applicable.
